# Effectiveness and safety of morinidazole in the treatment of pelvic inflammatory disease: A multicenter, prospective, open-label phase IV trial

**DOI:** 10.3389/fmed.2022.888186

**Published:** 2022-08-03

**Authors:** Ting Zhou, Ming Yuan, Pengfei Cui, Jingjing Li, Feifei Jia, Shixuan Wang, Ronghua Liu

**Affiliations:** ^1^Department of Obstetrics and Gynecology, Tongji Hospital, Tongji Medical College, Huazhong University of Science and Technology, Wuhan, China; ^2^Department of Obstetrics and Gynecology, Liuzhou Worker's Hospital, Liuzhou, China; ^3^Department of Obstetrics and Gynecology, Panjin Central Hospital, Panjin, China

**Keywords:** morinidazole, pelvic inflammatory disease, anaerobes, levofloxacin, safety

## Abstract

**Background:**

Antimicrobial resistance to metronidazole has emerged after several decades of worldwide use of the drug. The purpose of this study was to evaluate the effectiveness, safety and population pharmacokinetics of morinidazole plus levofloxacin in adult women with pelvic inflammatory disease (PID).

**Methods:**

Patients in 30 hospitals received a 14-day course of 500 mg intravenous morinidazole twice daily plus 500 mg of levofloxacin daily. A total of 474 patients were included in the safety analysis set (SS); 398 patients were included in the full analysis set (FAS); 377 patients were included in the per protocol set (PPS); 16 patients were included in the microbiologically valid (MBV) population.

**Results:**

The clinical resolution rates in the FAS and PPS populations at the test of cure (TOC, primary effectiveness end point, 7–30 days post-therapy) visit were 81.91 and 82.49% (311/377), respectively. There were 332 patients who did not receive antibiotics before treatment, and the clinical cure rate was 82.83%. Among 66 patients who received antibiotics before treatment, 51 patients were clinically cured 7–30 days after treatment, with a clinical cure rate of 77.27%. The bacteriological success rate in the MBV population at the TOC visit was 87.5%. The minimum inhibitory concentration (MIC) values of morinidazole for use against these anaerobes ranged from 1 to 8 μg/mL. The rate of drug-related adverse events (AEs) was 27.43%, and no serious AEs or deaths occurred during the study.

**Conclusions:**

The study showed that treatment with a 14-day course of intravenous morinidazole, 500 mg twice daily, plus levofloxacin 500 mg daily, was effective and safe. The results of this study were consistent with the results of a phase III clinical trial, which verified the effectiveness and safety of morinidazole.

## Introduction

PID is a common acute inflammatory disease associated with the upper reproductive tract of women, and this condition includes endometritis, salpingitis, tubal and ovary abscess, and pelvic peritonitis (1). PID is mainly caused by the upward spread of lower genital tract infection or by a sexually transmitted infection; it is mainly observed in young sexually mature women, and the most frequent age of onset is 20~35 years old (2, 3). According to some epidemiological data in China, the incidence rate of PID among adult women who are sexually active is 2% or higher. Untreated or inadequately treated PID can have serious clinical consequences, including infertility, ectopic pregnancy, and chronic pelvic pain. Early diagnosis and broad-spectrum antibacterial therapy are important to reduce the risk of both short-term and long-term complications (4–6). In addition, the risks of PID also include a possible association with ovarian cancer (7).

Antibiotic therapy is the basis of PID therapy. The treatment regimens for PID should include empiric broad-spectrum therapy to cover a wide range of pathogens, but the optimal treatment regimens still have not been determined. Previous studies have confirmed that PID is caused by various microorganisms, including sexually transmitted organisms, such as *Chlamydia trachomatis* and *Neisseria gonorrhoeae*, as well as microorganisms that are present in the vaginal flora. *Bacteroides fragilis* is the most prevalent anaerobe in abdominal and pelvic infections. The significant regional changes in the antibiotic sensitivity that are associated with anaerobes directly lead to poor results. Some antibiotic regimens that were recommended in the 1970s−80s are no longer suitable for the empiric treatment of PID, such as clindamycin, cefoxitin, cefotetan, and fluoroquinolones (including moxifloxacin) (8–13). Metronidazole is included in the regimens that are recommended for targeting anaerobic bacteria. Antimicrobial resistance to metronidazole has emerged after several decades of worldwide use of the drug. In addition, systemic metronidazole treatment is associated with several adverse reactions, including gastrointestinal discomfort and adverse events associated with the nervous system (14–18). One goal of the use of the new nitroimidazoles is to minimize the side effects and improve patient adherence to treatment.

Morinidazole is a new generation of nitroimidazole drugs. The China Food and Drug Administration (CFDA) approved the use of morinidazole in February 2014. Morinidazole has the following characteristics: (1) Its antibacterial activity is higher *in vitro* than those of metronidazole, ornidazole, and other nitroimidazoles, (2) Its high water solubility prevents it from easily penetrating the blood–brain barrier, so the incidence of adverse reactions in the central nervous system is lower than those observed for metronidazole, ornidazole, and other nitroimidazoles, (3) The main metabolic pathway does not go through the CYP system, unlike the pathway involved in the metabolism of metronidazole, ornidazole, and other nitroimidazoles. Therefore, there is little influence of morinidazole use on liver function, and there is less drug interaction. The results of a phase III clinical trial showed that morinidazole had slightly better efficacy than that of ornidazole and significantly better efficacy than that of metronidazole for anaerobes isolated in clinical tests. The results of pharmacodynamic tests *in vivo* and *in vitro* indicated that the antimicrobial activity of morinidazole against isolated pathogenic anaerobes was stronger than or equal to those of metronidazole, tinidazole, and ornidazole (19).

This paper describes a multicenter, prospective, open-label phase IV trial (NCT03391440, clinical trial registered on ClinicalTrials.gov) evaluating the effectiveness, safety and population pharmacokinetics of morinidazole in the treatment of adult females with PID.

## Methods

### Design scheme

This was a multicenter, prospective, and open-label trial conducted in mainland China from February 2017 to July 2019 (September 2016 to December 2018 on ClinicalTrials.gov were estimated when registering). The trial was performed in strict accordance with the Declaration of Helsinki and the Good Clinical Practice Guidelines. Ethical approval was obtained from the Huazhong University of Science and Technology, Tongji Medical College Affiliated Tongji Hospital Medical Ethics Committee (2014S00156). We obtained full written informed consent from all of the participants before the trial was performed.

According to the results of the phase III trial of morinidazole, the sample size was estimated to be 375 based on an α-value of 2.5% (one-sided) and a power of 80%. Assuming an 80% validity rate, 469 women were planned to be included in the study.

### Patients

The study enrolled women who were aged between 18 and 65 years old and who had PID (including endometritis, tubal phlogistic, oviduct ovarian abscess, pelvic peritonitis, etc.). The diagnosis of PID was based on the following symptoms: sexually active women or other patients with the risk of sexually transmitted infections; women with obvious lower abdominal tenderness, uterine tenderness, and/or adnexal and/or cervical motion tenderness on a biannual vaginal examination, accompanied by at least one of the following signs [from the China Guidelines for Diagnosis and Treatment of Pelvic Inflammatory Diseases (Revised Edition), 2014] (20): (A) pyrexia (axillary temperature ≥37.8°C); (B) mucopurulent cervical or vagina discharge; (C) an elevated white blood cell (WBC) count in vaginal discharge; (D) an elevated erythrocyte sedimentation rate; (E) an elevated C-reactive protein level; (F) cervical *Chlamydia trachomatis* infection confirmed by laboratory examination; and (G) a WBC count of ≥10×10^9^/L. Patients with an allergy to nitroimidazole, patients who had antibiotic therapy for 3 days within the last week, patients with any condition likely to require surgery, patients with impaired liver or renal function, patients who received an abortion within the last month, or patients with severe systemic diseases likely to affect the therapy (e.g., cardiovascular abnormalities, severe neuropathy, or epilepsy) were excluded from this study.

### Intervention

All participants received morinidazole (500 mg intravenous, twice daily for 14 days). Because of the diverse microflora involved in PID, levofloxacin was also administered to the participants. An intravenous infusion of 500 mg levofloxacin was administered once daily for the first week, followed by the oral administration of 500 mg levofloxacin tablets once daily for the second week.

The morinidazole and levofloxacin injections and levofloxacin tablets were provided free of charge by Shanghai Hansoh Biomedical Technology Co., Ltd. The morinidazole package was a 100 mL bottle containing 500 mg of morinidazole and 900 mg of sodium chloride. The batch No. used was NMPA Approval No. H20140022. The levofloxacin injection was produced by Jiangsu Hansoh Pharmaceutical Group Co., Ltd. The package was a 100 mL bottle containing 500 mg of levofloxacin and 900 mg of sodium chloride. The batch No. used was NMPA Approval No. H20041833. The levofloxacin tablets were produced by Jiangsu Hengrui Pharmaceutical Co., Ltd. The package contained 0.5 g/tablet. The batch No. used was NMPA Approval No. H20066387.

Patients attended the following study visits: a pre-treatment visit (3 days before the initiation of the study drug), an in-therapy visit (day 8), a first day post-therapy visit, and a TOC visit. The microbiology assessments were performed on cervical secretions that were taken from the pre-treatment visit, the first day post-therapy visit, and the TOC visit. The safety evaluations included patient compliance assessments, physical examinations and clinical laboratory assessments, which were performed at the pre-treatment visit, the in-therapy visit (day 8), the first day post-therapy visit and the TOC visit.

### Population pharmacokinetic study

According to the results of an analysis of the classification of the study population, a total of 70 subjects were enrolled in pharmacokinetic analysis subsets (PKSS). Participation in the population pharmacokinetic study was voluntary after full informed consent was obtained, and the subjects were randomly assigned to five groups (using Central stochastic system IWRS).

To ensure the accuracy of the blood sampling time of the population pharmacokinetic subjects, the infusion time of morinidazole was 45 ± 5 min, the interval between the first dose and the second dose on the first day was 8 h ± 10 min, and the interval between the first dose on the first day and the first dose on the second day was 24 h ± 30 min. According to the population requirements for the pharmacokinetic study, 70 subjects were randomly divided into 5 groups. Four blood samples were collected from each subject (3 mL each). The blood sampling time points are shown in Table 1.

**Table 1:** Blood sampling time points.

**Groups**	**Different blood sampling time**			
	**points after first infusion**			
	**A**	**B**	**C**	**D**
1	0.25 h ± 2 min	0.75 h ± 5 min	2.75 h ± 5 min	9.25 h ± 10 min
2	0.5 h ± 2 min	0.75 h ± 5 min	4.75 h ± 5 min	9.5 h ± 10 min
3	0.75 h ± 5 min	1.25 h ± 5 min	8.75 h ± 10 min	12.75 h ± 15 min
4	0.75 h ± 5 min	1.75 h ± 5 min	8.25 h ±10 min	16 h ± 15 min
5	0.75 h ± 5 min	8.25 h ± 10 min	10.75 h ± 10 min	24 h ± 30 min

### Analysis

The FAS group included all of the participants irrespective of the inclusion criteria. The PPS population was used for the effectiveness analysis and included all women who fulfilled the study inclusion criteria. The microbiologically valid (MBV) population (anaerobic cultures were performed in all subjects at the pre-treatment visit, but only subjects with positive anaerobic cultures were included in the MBV population) was included in the PPS population. The specimens from all of the patients were collected once during the screening period, the first day and 7–30 days after treatment for bacteriological examination. Each research center was responsible for the isolation, culture and identification of pathogenic bacteria.

The primary effectiveness indicator was the clinical response at the TOC visit in the PPS population. Clinical cure was defined as follows: an axillary temperature of ≤ 37.5°C, a WBC count of <10 × 10^9^/L, and a modified McCormack Scale score reduction of >90% compared with baseline values (according to the diagnosis and treatment guidelines of PID, the minimum diagnostic criteria for PID is uterine and/or adnexal tenderness. Corresponding with the diagnostic criteria, the criteria for clinical cure of PID include a modified McCormack Scale score reduction of >90% compared with the baseline value. The McCormack scale was used in this study to assess the severity of PID based on the self-perception of the subjects. The modified scale scores were taken before treatment, on day 1 in-therapy, day 8 in-therapy, the first day and 7–30 days after treatment), and a significant improvement or the disappearance of cervical or vaginal purulent secretions. The secondary effectiveness endpoints were as follows: (1) the clinical response on the first day post-therapy and (2) the bacteriological response (at the TOC visit and the first day post-therapy). Microbiological cure was defined as a negative result on the second cervical secretion culture.

### Statistical analysis

Statistical analysis was performed using SAS 9.4. The study mainly used descriptive statistics. The hypothesis test of the primary effectiveness indicator was one-sided and used an α = 0.025; all other statistical tests were two-sided and were performed at the 0.05 significance level.

## Results

### Patient and baseline characteristics

**Figure 1:**
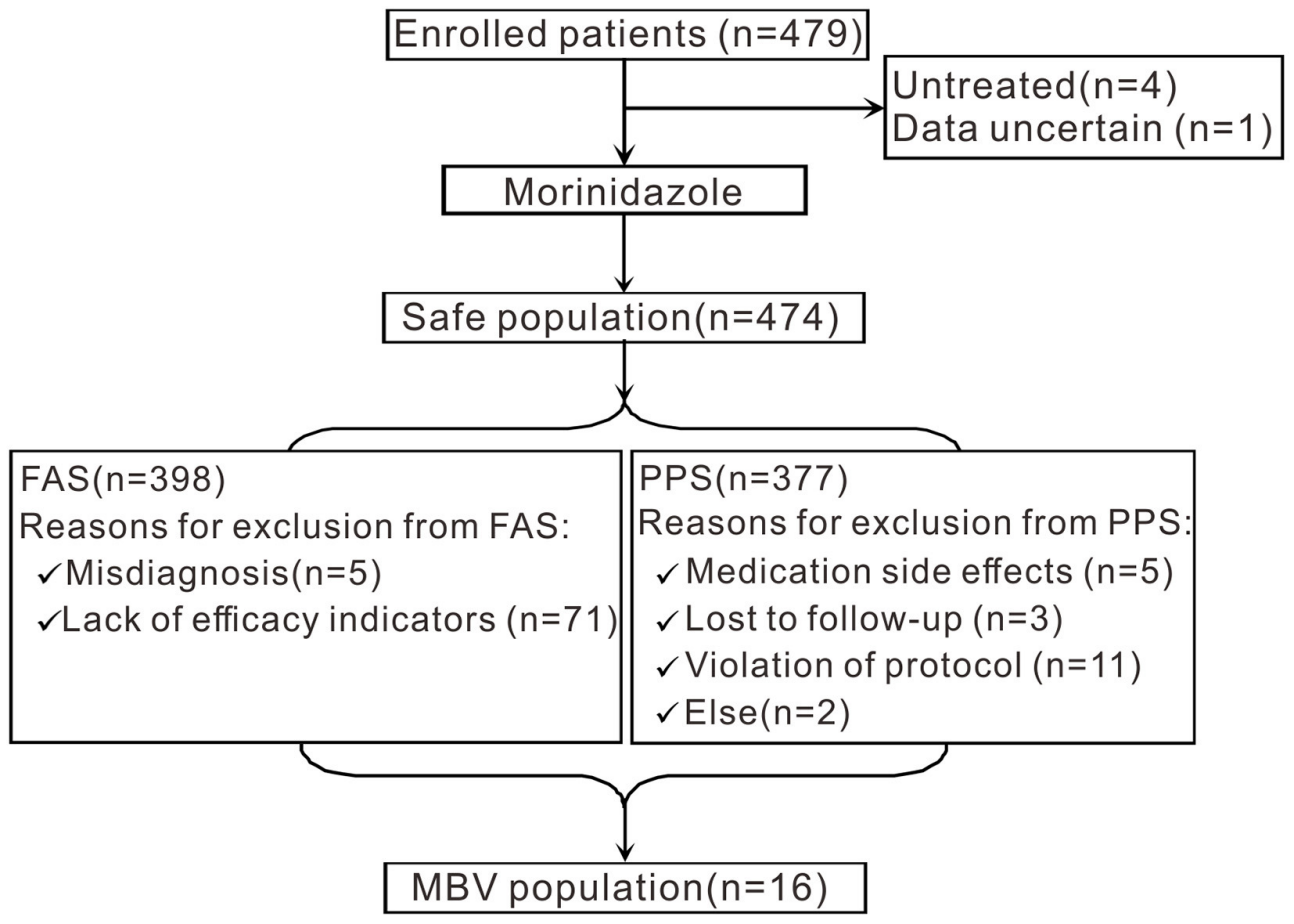
A flowchart of the study.

A flowchart of the study is shown in Figure 1. A total of 479 participants with PID from 30 centers were recruited for this study. Four patients were untreated, and 1 patient had ambiguous data. Therefore, 474 patients were included in the SS. A total of 398 patients were eligible for the FAS. Seventy-six patients were excluded from the FAS analyses (5 patients had a misdiagnosis, while the other 71 patients lacked effectiveness indicators). A total of 377 patients were eligible for the PPS analyses. Twenty-one patients were excluded from the PPS; among them, 5 patients developed medication side effects and 3 patients were lost to follow-up, and protocol violations occurred in 11 patients. Two patients were excluded for other reasons. Sixteen patients were included in the MBV population.

The demographic and baseline characteristics are shown in Table 2. The age of the subjects ranged from 18 to 62 years old, basically covering the whole range from youth to old, which is also the main demographic of PID patients. The duration from the onset of PID is as short as 1 day up to more than a year, representing acute, subacute, and chronic PID. As shown in Table 2, we observed that weight and height, which ranged from low to high, are good proxies for the different physical characteristics of the entire female population. The proportion of subjects with allergy history and drug history was 11.81 and 18.34%, respectively, and the proportion who had previously used antibiotics was 16.58%.

**Table 2:** Demographic and baseline characteristics.

**Characteristic**	**Morinidazole (*****N*** = **398)**			
	*N* (*N* miss)^*^	Mean ±SD^*^	M (Q_1_~Q_3_)^*^	Min~Max^*^
Age (years)	398 (0)	36.71 ± 8.94	36.00 (30.00 43.00)	18.00 62.00
Height (cm)	398 (0)	158.45 ± 5.36	158.00 (155.00 162.00)	140.00 174.00
Weight (kg)	398 (0)	55.87 ± 8.97	54.00 (49.00 60.00)	37.50 110.00
Duration of PID (days)	395 (3)	13.39 ± 35.76	3.00 (1.00 8.00)	1.00 386.00
Modified McCormack Scale score	398 (0)	8.44 ± 5.78	7.00 (4.00 11.00)	1.00 32.00
	**Yes**		**No**	
Allergy history, *n* (%)	47 (11.81%)		351 (88.19%)	
Medication history, *n* (%)	73 (18.34%)		325 (81.66%)	
Antibiotics before treatment *n* (%)	66 (16.58%)		332 (83.42%)	

* *N (N miss): Number (the number of subjects missing in the statistical analysis); Mean ± SD: Mean ± standard deviation; M (Q1~Q3): Percentile (numbers from 25 to 75); Min~Max: Minimum to maximum*.

### Effectiveness

#### Clinical response

Based on the clinical success criteria, the clinical resolution rate in the PPS population at the primary efficacy end point (TOC) was 82.49% (311/377). The clinical resolution rates for the FAS population were consistent with those for the PPS population. Among the 332 patients who did not receive antibiotics before treatment, 275 patients were clinically cured 7–30 days after treatment, and the clinical resolution rate was 82.83% (95% CI: 78.34–86.73%). Among the 66 patients who received antibiotics before treatment, 51 patients were clinically cured 7–30 days after treatment, with a clinical resolution rate of 77.27% (95% CI: 65.30–86.69%). According to the final results, the clinical resolution rate was less than 70% on the first day post-therapy, while the final clinical resolution rate reached 82.83%, which was considered to be related to the characteristics of the drug itself. Even in the patients who had previously taken other antibiotics with poor efficacy, the overall clinical effectiveness of morinidazole was satisfactory (Table 3).

**Table 3:** Clinical responses of the PPS and FAS populations at the TOC visit and the first day post-therapy visit.

**Responses**		**FAS (*n* = 398)**	**PPS (*n* = 377)**
TOC	Clinical resolution	326 (81.91%)	311 (82.49%)
	Clinical failure	72 (18.09%)	66 (17.51%)
	95% CI for Clinical resolution	(77.77, 85.57)%	(78.27, 86.19)%
First day	Clinical resolution	270 (67.84%)	263 (69.76%)
post-therapy	Clinical failure	128 (32.16%)	114 (30.24%)
	95% CI for Clinical resolution	(63.00, 72.41)%	(64.85, 74.36)%

#### Bacteriological response

All patients had culture samples taken from the cervical canal. Due to the small number of strains extracted in a single trial, to improve the reliability of the analysis, we analyzed the anaerobic strains extracted from two clinical trials together. Eighty-one strains of anaerobes were isolated from 43 patients (including 27 patients from another trial of morinidazole in patients with appendicitis, NCT03380793, clinical trial registered on ClinicalTrials.gov). In the study of patients with appendicitis, samples were obtained from surgically removed appendix tissue or from abdominal pus. Samples from both studies were sent to the same testing unit for MIC testing. The purpose of bacteriological research was to evaluate whether clinical isolates were susceptible to morinidazole. The bacteriological success rate in the MBV population at the TOC visit was 87.5% (14/16) (Table 4). The most commonly identified anaerobes were *Bacteroides fragilis* (*n* = 19), *Finegoldia magna* (*n* = 5), *Bacteroides thetaiotaomicron* (*n* = 6), *Prevotella bivia* (*n* = 12), and Lactobacillus (*n* = 6) (Table 5). Most of the tested strains were sensitive to morinidazole. The minimum inhibitory concentration (MIC) values for morinidazole ranged from 1 to 8 μg/mL and were very close to those of ornidazole and metronidazole (Table 5).

**Table 4:** Bacteriological success in the MBV population at the TOC visit.

**Visit**	**Bacteriological reaction**		
	**(Morinidazole**, ***N*** = **16)**		
	**Bacteriological**	**Bacteriological**	**95% CI**
	**success**	**failure**	
First day post-therapy	12 (75.00%)	4 (25.00%)	(47.62, 92.73)%
TOC	14 (87.50%)	2 (12.50%)	(61.65, 98.45)%

**Table 5:** The antibacterial activity (MIC) of morinidazole (compared with those of ornidazole and metronidazole) against anaerobes *in vitro*.

**Strains**	**Drug**	**MIC (**μ**g/ml)**		
		**MIC_50_**	**MIC_90_**	**MIC range**
Bacteroides fragile	Morinidazole	2	4	1–8
(*n* = 19)	Ornidazole	0.5	2	0.5–4
	Metronidazole	1	4	0.5–4
Finegoldia magna	Morinidazole	2	2	2–2
(*n* = 5)	Ornidazole	1	2	1–2
	Metronidazole	1	2	1–2
Bacteroides thetaiotaomicron	Morinidazole	2	8	1–8
(*n* = 6)	Ornidazole	1	1	0.5–1
	Metronidazole	1	2	0.5–2
Prevotella bivia	Morinidazole	1	1	1–1
(*n* = 12)	Ornidazole	0.5	0.5	0.5–0.5
	Metronidazole	0.5	1	0.5–1
Lactobacillus	Morinidazole	1	1	1–1
(*n* = 6)	Ornidazole	0.5	0.5	0.5–0.5
	Metronidazole	0.5	1	0.5–1

MIC, minimum inhibitory concentration; MIC_50_, minimum inhibitory concentration required to inhibit the growth of 50% of the tested strains; MIC_90_, minimum inhibitory concentration required to inhibit the growth of 90% of the tested strains.

### Improved McCormack scale score

In the FAS, the modified McCormack scale scores gradually decreased from day 8 in therapy to the first day after treatment and until 7–30 days after treatment, with reductions of 5.65 ± 4.43, 7.99 ± 5.86, and 8.08 ± 5.95, respectively. The modified McCormack scale scores at these three time points were compared with those obtained at baseline, and all of the differences were significant (*P* < 0.0001).

### Population pharmacokinetic parameters

**Figure 2:**
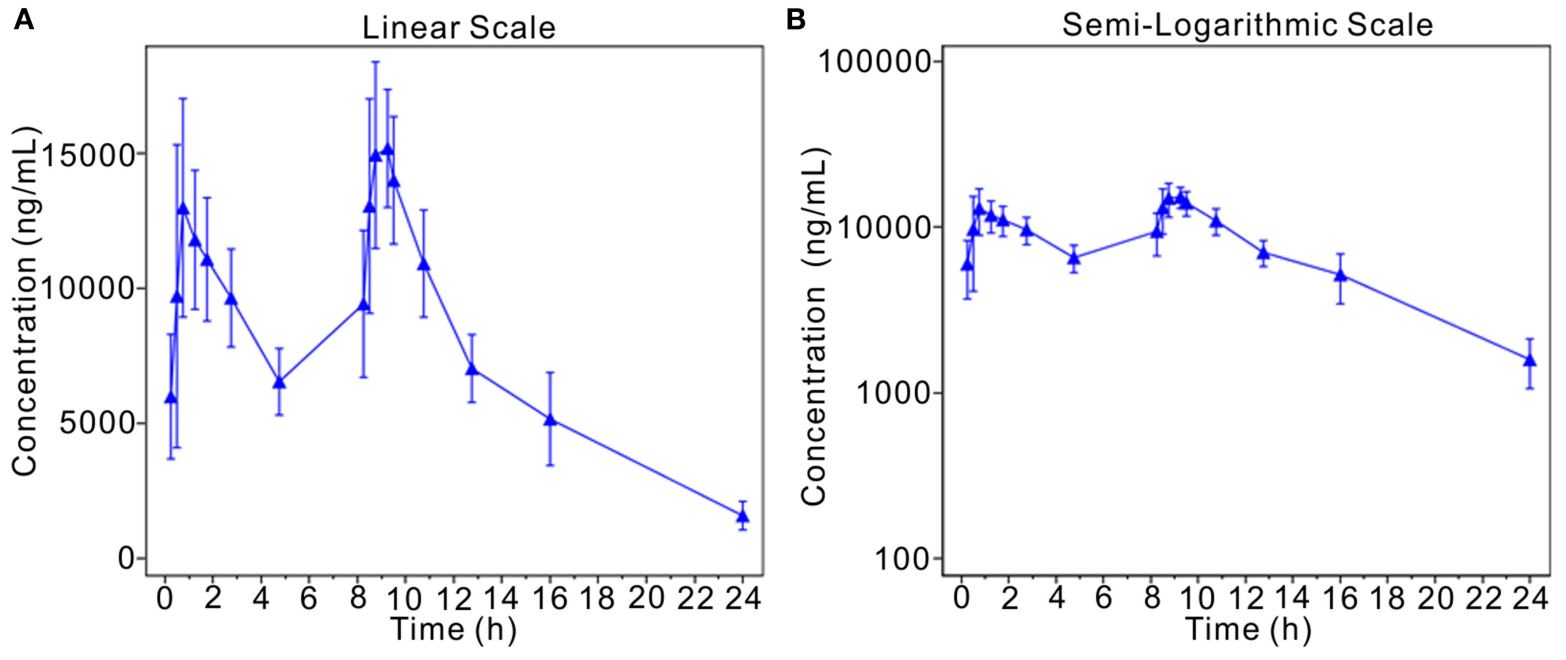
Mean plasma concentration-time curve (linear and semilogarithmic). The average drug concentration-time curves (**A**, linear; **B**, semilogarithmic).

This analysis assesses the blood drug concentrations of the subjects based on PKSS. Descriptive statistics were calculated for the blood drug concentrations of the subjects at the planned blood collection time, and the average drug concentration-time curves (linear and semilogarithmic) were drawn. As stated in the report, approximately 0.75 h and 9.25 h after the start of the first infusion, the average blood concentration of morinidazole was approximately 13,000 ng/mL, where its concentration peaked, and the concentration observed after the second infusion was slightly higher than the first concentration. These data suggested that morinidazole has stable pharmacokinetics in the population (Figure 2).

### Safety

In the FAS, the incidence of AEs was 44.73% (212/474), and the incidence of drug-related AEs was 27.43% (130/474). Most of the events were of mild to moderate severity. The most common drug-related AEs were nausea (5.91%), dizziness (3.80%), abdominal discomfort (2.32%), vomiting (2.11%), pruritus (1.90%), epigastric pain (1.69%), headache (1.69%), a decreased white blood cell count (1.69%), diarrhea (1.48%), flatulence (1.27%), and elevated blood creatinine levels (1.05%). Serious AEs occurred in 6 cases (1.27%), including somatization disorder, ovarian serous adenocarcinoma, adjustment disorder, gonorrhoeae-infected PID, endometrial polyp, and chronic gastroenteritis. Among them, 2 cases (somatization disorder and adjustment disorder) were judged to be probably unrelated to drugs, while the rest were unrelated. No serious drug-related AEs or deaths occurred during the study (Table 6).

**Table 6:** Occurrences of adverse events, including the most common drug-related adverse events (>1%).

**Occurrences of adverse events (*****N*** = **474)**	
Any adverse event, *n* (%)	212 (44.73)
Any drug-related adverse event, *n* (%)	130 (27.43)
Nausea	28 (5.91)
Dizziness	19 (3.80)
Abdominal discomfort	11 (2.32)
Vomiting	10 (2.11)
Pruritus	9 (1.90)
Epigastric pain	8 (1.69)
Headache	8 (1.69)
Decreased white blood cell count	8 (1.69)
Diarrhea	7 (1.48)
Flatulence	6 (1.27)
Elevated blood creatinine	5 (1.05)
Serious adverse event, *n* (%)	6 (1.27)
Serious drug-related adverse event, *n* (%)	0 (0.00)
Death, *n* (%)	0 (0.00)

## Discussion

PID is often a mixed infection. If there is an anaerobic infection and the patient is older, the patient is prone to multiple recurrences, usually accompanied by abscess formation, such that treatment is more problematic (20, 21). PID patients usually exhibit long-term sequelae, and some scholars believe that this is due to the formation of scars and adhesions during the healing of infected tissues. There are different views on the exact mechanism. For example, it is unknown whether it is related to the standard and rational use of antibiotics, or whether it is related to insufficient targeting of anaerobic bacteria by the antibiotics or resistance to antibiotics. However, the role of anaerobic bacteria in PID is still controversial.

Due to the difficulty associated with culturing anaerobic bacteria, the overall clinical detection rate was not high in previous studies. The rate of the detection of anaerobic bacteria in the genital tract is higher in patients with acute PID, especially for vaginal disease-related anaerobic bacteria (22–25). Even so, the isolation and cultivation of anaerobes is still difficult and requires a long time, and this process may not be able to guide clinical treatment within a reasonable amount of time, so anti-anaerobic drugs and anti-pathogenic drugs are commonly used in combination. The CDC currently recommends considering the addition of metronidazole in all outpatient treatments of PID patients and in patients who have BV, trichomoniasis or who were exposed to recent uterine instrumentation (26, 27).

The clinical resolution rate of the phase IV clinical trial was close to that achieved in the relevant treatment regimens reported in the previous literature (28–30). There were some differences in the combination of drugs used. In addition, the actual infection rate of anaerobes was relatively unclear, so a stratified analysis and other methods should be considered in future studies. Individualized medication should be given to patients who are highly suspected of having an anaerobic infection to improve the clinical resolution rate.

The trial showed that morinidazole was safe and well-tolerated. The drug-related AEs mainly occurred in the digestive system and nervous system, which were also common drug-related AEs observed in previous clinical practice (31, 32), and there were no unexpected drug-related AEs. The bacteriological evaluation showed that morinidazole had a similar antibacterial spectrum for targeting the tested anaerobic bacterial strains to those observed for ornidazole and metronidazole. Morinidazole had obvious antibacterial activity against the tested anaerobic bacterias, and the antibacterial effect against most of the tested strains was sensitive.

The phase IV trial had a larger sample size than did the phase III trial. Moreover, this trial involved 30 hospitals, covering not only most geographical areas in China but also people of different ages and ethnic groups. Our results represent the basic situation of PID in secondary and tertiary hospitals in China. The main purpose of phase IV clinical trials is to verify the effectiveness and safety of morinidazole, so we chose levofloxacin, which has a weak effect on anaerobic bacteria, as the combination drug. The trial protocol used in the phase IV trial was more consistent with the clinical situation and the requirements of the guidelines than the protocol used in the phase III trial, so we consider this study to have higher external validity. In addition, we used the same data collection format in all hospitals to improve consistency and reliability. Finally, all pathogenic bacteria were uniformly transported to the central laboratory for reverification and MIC determination. The MICs of the anaerobes were evaluated. The strains were kept in the central laboratory for inspection by a supervisory department, and the information collected from this study is important for implementing more relevant and effective intervention strategies for each area. Furthermore, because it is inconvenient to perform a gynecological examination during menstruation, we conducted an effectiveness evaluation 7 and 30 days after the end of treatment to rule out this situation to provide more objective data.

Some limitations of the study deserve consideration. First, 84 subjects dropped out (17.54%) from the trial. The main reasons for the loss of participants were early withdrawal because of adverse events and self-withdrawal or loss of patients to follow-up. However, the results still fall into the category of statistical acceptability. When possible, various measures should be taken to reduce the occurrence of these situations. Additionally, we noticed that the clinical resolution rate was slightly lower than that observed in the phase III trial. Whether the presence of anaerobic bacteria is universal in the pathogenesis of PID is unknown. There may be other confounding factors. Beyond these speculations, it is unknown whether the results were related to the different combinations of drugs used. More studies should be performed to explore the results. Additionally, the phase III and phase IV trials were conducted at different periods and in different settings and were not head-to-head studies; thus, it may not be easy to compare them directly. Finally, although the cervical secretions of each patient were collected and examined, the culture rate of anaerobes was generally low due to the high difficulty of culturing anaerobes.

This study was a multicenter, prospective, open-label phase IV trial evaluating the effectiveness, safety and population pharmacokinetics of morinidazole in the treatment of adult females with PID. The results showed that treatment with a 14-day course of intravenous morinidazole, 500 mg twice daily, plus levofloxacin 500 mg daily, was effective. At the same time, the bacteriological success rate was satisfactory, and there were few side effects. The results of this study were basically consistent with the results of a phase III clinical trial, which verified the effectiveness and safety of morinidazole. Therefore, we believe that morinidazole is a new drug option for patients with PID.

## Data availability statement

The raw data supporting the conclusions of this article will be made available by the authors, without undue reservation.

## Ethics statement

The studies involving human participants were reviewed and approved by the Ethics Committee of the Tongji Hospital, Tongji Medical College, Huazhong University of Science and Technology approved the study. The patients/participants provided their written informed consent to participate in this study. Written informed consent was obtained from the individual(s) for the publication of any potentially identifiable images or data included in this article.

## Author contributions

TZ, MY, SW, and RL contributed to the conceptualization of the study. MY, JL, and FJ assisted in data collection. TZ and RL are responsible for the supervision of the clinical trial. RL has access to the final trial dataset. All authors have read and approved the manuscript for publication.

## Funding

This study received funding from Jiangsu Hansoh Pharmaceuticals Limited. The funder was not involved in the study design, collection, analysis, interpretation of data, the writing of this article, and the decision to submit it for publication.

## Conflict of interest

The authors declare that the research was conducted in the absence of any commercial or financial relationships that could be construed as a potential conflict of interest.

## Publisher's note

All claims expressed in this article are solely those of the authors and do not necessarily represent those of their affiliated organizations, or those of the publisher, the editors and the reviewers. Any product that may be evaluated in this article, or claim that may be made by its manufacturer, is not guaranteed or endorsed by the publisher.
